# Tumor-Induced Cardiac Dysfunction: A Potential Role of ROS

**DOI:** 10.3390/antiox10081299

**Published:** 2021-08-18

**Authors:** Priyanka Karekar, Haley N. Jensen, Kathryn L. G. Russart, Devasena Ponnalagu, Sarah Seeley, Shridhar Sanghvi, Sakima A. Smith, Leah M. Pyter, Harpreet Singh, Shubha Gururaja Rao

**Affiliations:** 1 Department of Physiology and Cell Biology, The Ohio State University, Columbus, OH 43210, USA; karekarp223@gmail.com (P.K.); jensen.315@buckeyemail.osu.edu (H.N.J.); devasena.ponnalagu@osumc.edu (D.P.); shridhar.sanghvi@osumc.edu (S.S.); 2 Institute for Behavioral Medicine Research, Departments of Psychiatry and Behavioral Health & Neuroscience, The Ohio State University, Columbus, OH 43210, USA; klgrussart@gmail.com (K.L.G.R.); leah.pyter@osumc.edu (L.M.P.); 3 Department of Pharmaceutical and Biomedical Sciences, Raabe College of Pharmacy, Ohio Northern University, Ada, OH 45810, USA; s-seeley.1@onu.edu; 4 Division of Cardiovascular Medicine, Ohio State University Wexner Medical Center, Columbus, OH 43210, USA; sakima.smith@osumc.edu

**Keywords:** heart failure, cancer, mitochondria, reactive oxygen species, Hippo–Yorkie pathway

## Abstract

Cancer and heart diseases are the two leading causes of mortality and morbidity worldwide. Many cancer patients undergo heart-related complications resulting in high incidences of mortality. It is generally hypothesized that cardiac dysfunction in cancer patients occurs due to cardiotoxicity induced by therapeutic agents, used to treat cancers and/or cancer-induced cachexia. However, it is not known if localized tumors or unregulated cell growth systemically affect heart function before treatment, and/or prior to the onset of cachexia, hence, making the heart vulnerable to structural or functional abnormalities in later stages of the disease. We incorporated complementary mouse and *Drosophila* models to establish if tumor induction indeed causes cardiac defects even before intervention with chemotherapy or onset of cachexia. We focused on one of the key pathways involved in irregular cell growth, the Hippo–Yorkie (Yki), pathway. We used overexpression of the transcriptional co-activator of the Yki signaling pathway to induce cellular overgrowth, and show that Yki overexpression in the eye tissue of *Drosophila* results in compromised cardiac function. We rescue these cardiac phenotypes using antioxidant treatment, with which we conclude that the Yki induced tumorigenesis causes a systemic increase in ROS affecting cardiac function. Our results show that systemic cardiac dysfunction occurs due to abnormal cellular overgrowth or cancer elsewhere in the body; identification of specific cardiac defects associated with oncogenic pathways can facilitate the possible early diagnosis of cardiac dysfunction.

## 1. Introduction

Cancer is the second leading cause of death in the world [1] and in the United States, it accounts for 25% of deaths per year [2]. An estimated 50–80% of cancer patients develop cachexia, a syndrome involving loss of muscle and adipose tissue [3]. This condition often leads to a severely reduced quality of life due to symptoms including fatigue, anemia, prolonged inflammation, altered metabolism, and depression [3]. Nearly 30% of cancer-related deaths are caused by complications of cachexia, such as respiratory and heart failure [4]. Radiation and chemotherapy-induced cardiotoxicity in cancer patients has also been implicated in heart failure [5,6,7]; however, more recent studies suggest that cardiac dysfunction could also be a direct effect of tumor biology [8,9].

Cardiac cachexia is a multifactorial syndrome that is associated with heart dysfunction and eventual heart failure. It is caused by interacting inflammatory cytokines, lipolytic and proteolytic factors released by tumors and cachectic myocytes, neurohormones, and muscle catabolism [10]. Several studies have identified potential biomarkers for cancer-induced cardiac dysfunction; for example, one study showed that patients with lung, pancreatic, and gastrointestinal cancer had a high incidence of cardiac cachexia [11]. There have also been recent studies on cancer-induced cardiac cachexia in mice using adenocarcinoma and melanoma cells, to find involved biomarkers and mechanisms of action. One study showed an increase in matrix metalloproteases in cachectic cardiac muscle, implying its role in cardiac dysfunction [12]. In another study, cardiac wasting in cancerous mice was found to be associated with a disruption in antioxidant systems and reactive oxygen species (ROS) [13]. These studies point at a change in cardiac signaling molecules that leads to the activation of the ubiquitin-proteasome system or autophagy/mitophagy pathways in the heart causing cardiac cachexia [14]. However, there is not a clear understanding yet of how different types of tumors trigger different pathways and their differential effect on cardiac dysfunction. Nor it is known if cardiac dysfunction can occur before cachexia. The majority of the studies have focused on post-chemotherapy cardiac phenotypes of patients but not the direct effect of tumors on cardiac tissue and function. Thus, a detailed investigation of tumor-induced cardiac effects is required to understand the signaling mechanisms causing dysfunction of the heart in a tumor-specific manner.

The primary goal of this study was to understand the relationship between tumor biology and associated systemic cardiac dysfunction, and possible mechanisms involved in cancer-mediated heart failure using a genetically amenable *Drosophila melanogaster* model system. In addition, the study assessed the time of onset of cardiac dysfunction in mice by implanting breast cancer cells in adult female Balb/c mice where we observed an increase in the rate of ROS production in the heart in tumor-bearing mice. Finally, the study determined molecular pathways in specific tissues that cause cardiac dysfunction by expressing different oncogenes using the UAS-Gal4 system in *Drosophila* [15]. We explored in detail the oncogene-induced cardiac effects through the established tumor causing Yki oncogene in the Hippo pathway. We evaluated cardiac phenotypes using optical coherence tomography (OCT) and imaging for cardiac morphology. We also were able to rescue the phenotypes using antioxidant feeding, suggesting a role for ROS in tumor-induced cardiac effects. Our study is one of the first studies to show systemic cardiac phenotypes of tumors in a genetic model system.

## 2. Methods

### 2.1. Fly Stocks

Fly stocks were obtained from Bloomington *Drosophila* Stock Center (Bloomington, IN, USA) and incubated at 25 °C on jazz mix media (Fisher, Pittsburgh, PA, USA). The W^1118^ fly strain was used as the wild type (wt) fly. Oncogene overexpressing UAS (Upstream activating sequence) strains used were PI3K (*P[w[+mc] = UAS-Pi3K92E.CAAX]1,y [1]w[1118]*), RasV12 (w[*]; P[w[+mC] = UAS-Ras85D.V12]2), Hep^Act^ (w[*]; P[w[+mC] = UAS-Hep.Act]2) and *UAS-Yki* (gift from D. Pan lab, UT Southwestern, Dallas). Gal4 drivers used were GMR (w[*]; P[w[+mC] = GAL4-ninaE.GMR]12), eyeless (w[*]; P[w[+m*] = GAL4-ey.H]3-8) and dpp (w[*]; wg[Sp-1]/CyO; P[w[+mW.hs] = GAL4-dpp.blk1]40C.6/TM6B, Tb[1]).

Fly crosses were set up at 25 °C between UAS lines-RasV12, PI3K, Hep^Act,^ and the three Gal4 lines in all combinations. UAS-Yki flies were crossed with GMR-Gal4 flies to create a stable line of GMR-Gal4>UAS-Yki flies. Larvae and flies obtained from these crosses were kept at 29 °C and used for further experiments. Control larvae were obtained from crosses between the UAS lines and wt. GGal4 flies were used as controls for GMR-Gal4>UAS-Yki flies.

### 2.2. Tumor Induction and Heart Function in Mice

All animal experiments were approved by the Ohio State University Institutional Animal Care and Use Committees and carried out in accordance with the National Institutes of Health Guide for the Care and Use of Laboratory Animals (2014A00000093-R2, NRC, 2011). All efforts were made to minimize animal suffering and to reduce the number of mice used. This project consists of two treatment-balanced experimental replications. Under anesthetization (isoflurane vapors), a 5 mm subcutaneous incision was made medial to the 4th nipple, and 5 × 10^6^ 67NR breast cancer cells (in matrigel) or PBS for tumor-free controls were injected into the associated mammary fat pad of adult female Balb/c mice (as described in [16]; *n* = 8–9/group). Echocardiography of the control and tumor-bearing mice were carried out four weeks after the tumor cells’ injection (tumors ~1.5 cm diameter) as per established protocols [17,18,19]. Briefly, under 1.5% (*v/v*) isoflurane, echocardiography was performed on mice using Vevo 3100. B- and M-mode images were obtained, and data were analyzed to calculate left ventricular ejection fraction (LVEF) and LV fractional shortening.

### 2.3. Optical Coherence Tomography (OCT) Recordings

Flies were selected from each oncogenic cross for evaluating cardiac function by OCT. Flies were anesthetized using CO_2_ and placed on a thin layer of high vacuum grease (DOW Corning, Midland, MI, USA) to immobilize. The Telesto-II OCT system (Thorlabs, Munich, Germany) was used to obtain two-dimensional B-mode (at 76 kHz) and M-mode (at 5.5 kHz) images of the cardiac tubes, by placing the animals with their dorsal side facing the OCT probe. The cardiac tube was located and centered at the abdomen in B-mode, and then the system was switched to M-mode to record continuous cardiac cycles.

### 2.4. Cardiac Function Analysis

The recorded two-dimensional M-mode OCT traces were analyzed to quantify end-diastolic diameter (EDD) and end-systolic diameter (ESD) through the ThorImage software (Thorlabs, USA) [20]. Averages were taken for the EDD and ESD dimensions of each fly, and these dimensions were used to calculate the fractional shortening (FS) defined as [(EDD − ESD)/ESD] × 100. Heart rate (HR) was calculated by averaging the number of systoles in 5–10 frames of 1.83 s each. Stroke volume (SV) was calculated as EDV − ESV, and cardiac output (CO) was calculated as SV × HR. The arrhythmic index was calculated as the ratio of arrhythmias per minute over HR. An arrhythmic event was considered to occur when the distance between two systolic events (systolic interval) was larger than twice the average systolic interval. For mice, comprehensive echocardiography analysis was performed using Vevo 3100 (FUJIFILM Visual Sonics Inc., Toronto, ON, Canada) as described earlier [18,19,21]. Mice were euthanized by an overdose of CO_2_ followed by decapacitation as per the IACUC guidelines.

### 2.5. Measurement of Pericardin Fiber Thickness

Cardiac tubes were dissected from third instar larvae and fixed in ice-cold 4% (*w/v*) paraformaldehyde (PFA) and washed and permeabilized with 0.4% (*v/v*) Triton-X100. Tissues were blocked with 10% normal goat serums and stained overnight at 4 °C using mouse-anti Pericardin monoclonal antibody EC11, followed by a secondary antibody and DAPI. Z-stack images of each sample were captured at 0.5 µm. Fiji Image J software, (NIH, Bethesda, MD, USA) was used to find the diameters of 40 to 60 fibers per tissue sample, and data were combined from all samples of the same group and plotted as a frequency histogram with a bin size of 0.05 µm.

### 2.6. ROS Staining

Cardiac tubes of 3rd instar larvae were dissected and stained in dihydroethidium (DHE, Molecular Probes, Eugene, OR, USA), as described in earlier publications [20]. Tissues were fixed and then mounted on microscope slides to image within the next 20 min. Cardiac tubes were imaged using the Olympus FV1000 confocal microscope, (Olympus, Tokyo, Japan) using a 10× objective lens at 568 nm excitation wavelength.

### 2.7. Hemolymph ROS Measurement

Hemolymph was collected from 3rd instar larvae by tearing and spinning down the larvae. Mouse heart mitochondria were isolated and ROS was measured as described earlier [22,23,24]. ROS was measured on the Hitachi Fluorescence Spectrophotometer F-7100 as described previously [25]. Fluorescence was recorded with excitation at 560 nm and emission at 590 nm. We added 5 μg horseradish peroxidase (Sigma-Aldrich, St. Louis, MO, USA) to 2 mL ROS buffer [mmol/L, 20 Tris-HCl, 250 sucrose, 1 EGTA-Na_2_, 1 EDTA-Na_2_, pH 7.4 at 25 °C] in a cuvette, and fluorescence recording was started. We added 10 μM Amplex^®^ Red (Thermofisher, Waltham, MA, USA) after 1 min, followed by 25 μL hemolymph and 10 mM succinate (each after 1 min), and fluorescence was recorded for 15 min.

### 2.8. Antioxidant Feeding

Fly media with antioxidants was made by mixing 25 mg/mL l-ascorbic acid (Vitamin C (Sigma-Aldrich, St. Louis, MO, USA)), 1 mM α-tocopherol (Vitamin E (Sigma-Aldrich, St. Louis, MO, USA)), 1% (*w/v*) ubiquinone (CoQ10(Sigma-Aldrich, St. Louis, MO, USA)), or 1 mM reduced l-glutathione (GSH (Sigma-Aldrich, St. Louis, MO, USA)) in Jazz mix fly media. GMR and GMR>Yki flies were allowed to feed on antioxidant media after emerging into flies. Another set of the same flies were allowed to lay eggs and grow in antioxidant media. Adult flies from both sets were collected on day 7 for recording cardiac function.

### 2.9. Statistical Analysis

Data are reported as mean ± SEM. Student’s *t*-test, one-way ANOVA and Holm–Sidak test (multiple comparisons to control), or Student–Newman–Keuls test (multiple pairwise comparisons) were used to compare groups, with significance level *p* ≤ 0.05 (# *p* < 0.05, * *p* < 0.01, ## *p* < 0.005, ** *p* < 0.001).

## 3. Results

### 3.1. Tumorigenesis Causes Systemic Cardiac Dysfunction

**Figure 1:**
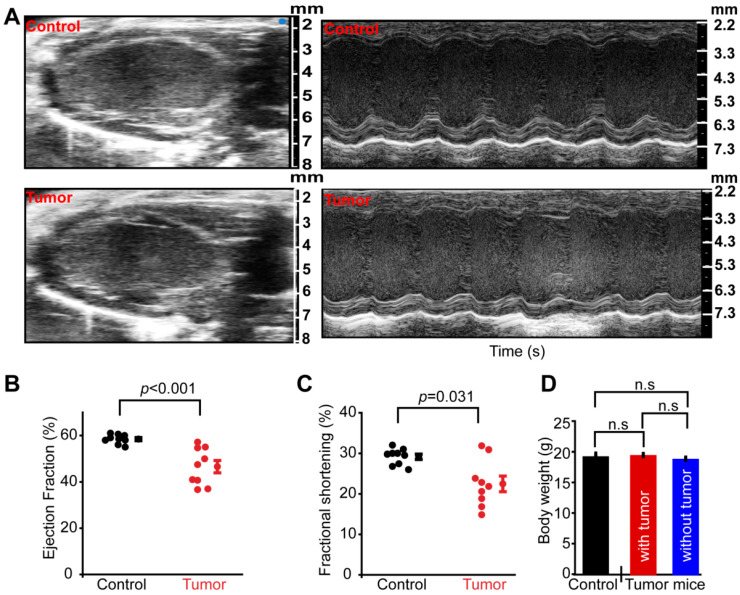
Tumors cause cardiac dysfunction in mice. Tumors were induced in C57Bl6 mice by injecting adenocarcinoma cells into mammary glands. After 4 weeks echocardiography was performed on all the mice. (**A**) Echocardiography images were obtained in B- (**left**) and M-mode (**right**). Images were obtained for mice with or without tumors; (**B**) Left ventricular ejection fraction significantly reduced in mice with tumors (*p* < 0.001, *n* ≥ 8); (**C**) Mice with tumor showed a lower left ventricular fractional shortening (*p* < 0.03, *n* ≥ 8); (**D**). The body mass of sham/control, tumor-bearing mice (given both with or after tumor excision). The body mass between sham and tumor groups (with or after tumor excision) was comparable. Statistical significance was calculated by Student’s *t*-test and ANOVA.

To investigate the effect of oncogenes on cardiac function in a mammalian model system without the intervention of therapeutics, we induced tumor growth in a mouse model and measured cardiac function. Mice were injected with adenocarcinoma cells in their mammary glands. After 4 weeks of tumor growth, we measured cardiac function using echocardiography. We observed a significant reduction in cardiac function as shown in Figure 1. Our echocardiography analysis (Figure 1A) showed left ventricular ejection fraction (LVEF, Figure 1B) and left ventricular fractional shortening (LVFS) (*n* ≥ 9) (Figure 1C) were reduced by 20 ± 5% and 22 ± 6%, respectively (*n* ≥ 8 mice in each group), indicating that the tumor in the mammary tissue caused a systemic effect on the heart causing reduced function. These cardiac dysfunction outcomes were similar to the cancer-cachexia model where male mice injected (*s.c.*) with adenocarcinoma cells presented reduced LVFS [26]. These results suggest that tumors can cause systemic cardiac dysfunction even before the use of any therapeutic interventions. It is to be noted that, as shown in Figure 1D, the body mass of animals was similar between sham and tumor groups whether the tumor-bearing mice were compared with or without tumor weight. This indicates the tumor-bearing mice did not have considerable body mass loss due to cachexia and cardiac phenotypes show before the onset of cachexia. Our results for the first time indicate that cardiac dysfunction is present in tumor-bearing mice before the onset of cachexia or any therapeutic intervention. However, the precise signaling pathway involved in cardiac dysfunction caused by tumors is not identified.

### 3.2. Overgrowth Causes Systemic Cardiac Dysfunction in Drosophila

**Figure 2:**
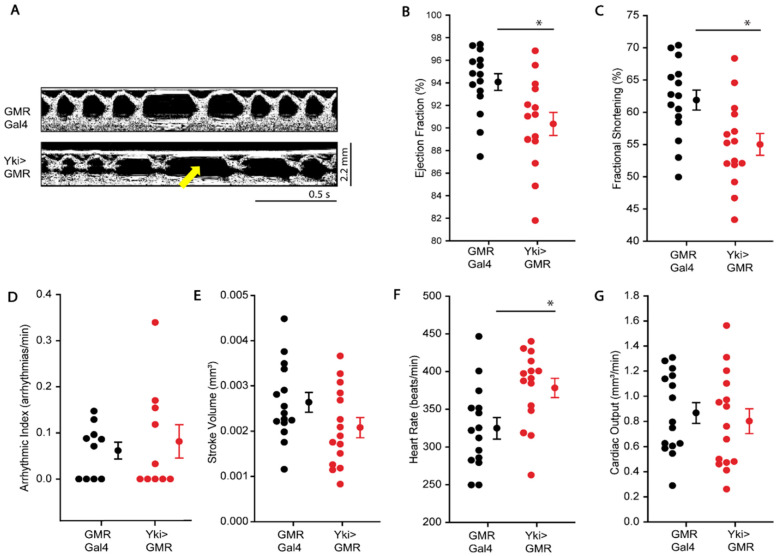
Cardiac function of 3–5 day old flies overexpressing Yki using GMR-Gal4 driver. (**A**) M-mode images of cardiac tubes of GMR>Yki and GMR-Gal4 control animals. (**B**–**G**) show EF, FS, AI, SV, HR, and CO, respectively, of GMR>Yki compared with GMR-Gal4 control. * *p* < 0.01; *n* ≥ 25 flies. Statistical significance was calculated by the Holm–Sidak test (multiple comparisons to control), and the Student–Newman–Keuls test (multiple pairwise comparisons).

To identify the putative signaling pathways involved in tumor-induced cardiac dysfunction we used the genetically amenable system *Drosophila melanogaster* by overexpressing oncogenes in imaginal discs and measuring heart function using optical coherence tomography (OCT) [20]. We chose a multitude of pathways (Table 1) such as the Yorkie pathway (Figure 2), PI3K (Supplemental Figure S1), RasV12 (Supplemental Figure S2), and Hep^Act^ (Supplemental Figure S3) in an unbiased manner. We observed altered cardiac function as measured by OCT in all the oncogenic backgrounds in various degrees (Table 1). All the mutants exhibit reduced ejection fraction/fractional shortening as observed in mice (Figure 1), and differences in cardiac output (Table 1). For example, Hep Activated (JNK) shows increased stroke volume, whereas it is decreased for PI3K kinase. Our results summarized in Table 1 indicate that not all oncogenes have the same effect on heart function and Drosophila presents a testable model to study these tumor-induced cardiac dysfunctions. We also measured cardiac fibrosis by staining for pericardin and quantifying the thickness of the pericardin fibers. We did not notice any difference in pericardin fiber thickness (Supplemental Figure S4) indicating that no structural changes as observed and reported in cachexia [27] are occurring in the cardiac tubes of flies overexpressing oncogenes for a given time span.

### 3.3. Effect of Overexpression of Yorkie-Induced Overgrowth on Cardiac Function

To decipher the mechanism of tumor-induced cardiac dysfunction, we chose the Yorkie (Yki) pathway for further analysis. We focused on Yorkie as we had a clear system where we could express the oncogene only in the eye in adult stages with GMR driver and study heart function. Yki, a homolog of the mammalian YAP, is a part of the Hippo (Hpo)-Yki signaling pathway [28]. This signaling pathway controls growth and regulates the size of organs through a cascade of kinases, which inhibits Yki/YAP, a transcriptional coactivator that promotes cell proliferation and inhibits apoptosis [29]. Overexpression of Yki in Drosophila larval eye imaginal discs is known to cause an overgrowth in adult eye size [30].

As shown in Figure 2, overexpressing Yki using GMR Gal4 driver, which results in severe overgrowth of the eye but not expressed in the heart [31], results in cardiac dysfunction. We observed significantly lower EF and FS in GMR>Yki larvae as compared to GMR-Gal4 control larvae (Figure 2B,C). HR was found to be increased in these larvae as well (Figure 2F). No differences were observed in SV and CO (Figure 2E,G). We noticed occasional arrhythmic events in both GMR-Gal4 and GMR>Yki larvae, but their calculated arrhythmic index was not significantly different (Figure 2A,D). These larvae also did not show signs of cardiac fibrosis (Supplemental Figure S4D).

### 3.4. Quenching Reactive Oxygen Species Rescues Cardiac Phenotypes

**Figure 3:**
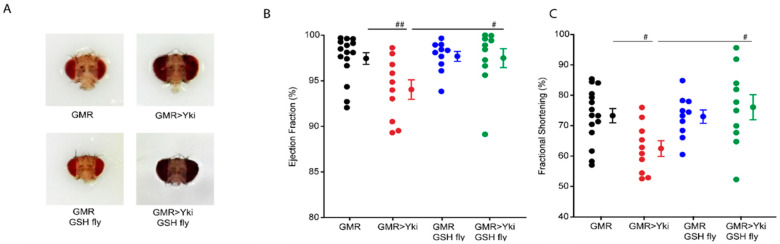
Heart function of 7-day-old flies overexpressing Yki using GMR-Gal4 driver, with glutathione supplementation from day 1–7. (**A**) Headshots of flies showing eyes with overgrowth. Note that GSH feeding does not reduce overgrowth; (**B**) Quantification of ejection fraction of flies; (**C**) Quantification of fractional shortening of flies. GSH feeding rescued EF and FS or GMR>Yki flies. # *p* < 0.05; ## *p* < 0.005, *n* ≥ 25 flies. Statistical significance was calculated by the Holm–Sidak test (multiple comparisons to control), and the Student–Newman–Keuls test (multiple pairwise comparisons).

One of the key factors involved in cardiac dysfunction is oxidative stress caused by dysregulated ROS production [21,32]. Therefore, we explored the possibility of rescuing cardiac dysfunction in Yki-overexpressing flies by reducing oxidative stress. We fed the Yki flies with antioxidants (GSH, Vitamin E, CoQ 10, and Vitamin C) from eclosion to day 7, and measured their cardiac function on day 7. Yki-overexpressing flies fed with antioxidants after eclosion and imaged in Day 7 showed an improvement of cardiac function. GSH feeding does not reduce the overgrowth of the eye (Figure 3A) but rescued cardiac ejection fraction (Figure 3B) and fractional shortening (Figure 3C). Feeding flies with vitamin E and CoQ 10 also rescued cardiac dysfunction but not overgrowth (Supplemental Figures S5 and S6). However, feeding Yki-overexpressing flies with vitamin C does not rescue heart function (Supplemental Figure S7). Our results indicate that selective antioxidant treatment to Drosophila-bearing tumors helps with their cardiac dysfunction.

### 3.5. Systemic Reactive Oxygen Species Increase in Yki-Overexpressing Flies

**Figure 4:**
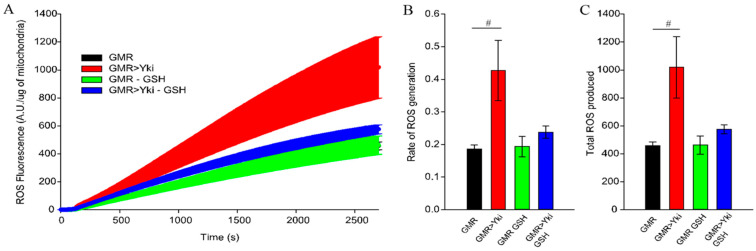
Overexpression of Yki results in mitochondrial ROS generation. ROS was measured in isolated mitochondria from control and Yorkie-overexpressing flies (GMR and GMR>Yki). Larvae were also fed with glutathione and mitochondria were isolated after 7 days of eclosion. (**A**) Graph representing ROS production for 45 min after addition of isolated mitochondria from GMR (black), GMR>Yki (red), GMR fed with GSH (green) and GMR>Yki fed with GSH (blue); (**B)** Bar graph representing a rate of ROS production; (**C**) Total ROS generation calculated from (**A**). # *p* < 0.05; *n* ≥ 25. (statistical significance was calculated by Student’s *t*-test).

Since we observed rescue of cardiac dysfunction by feeding flies with antioxidants, we tested whether Yorkie-overexpressing flies have increased systemic ROS compared to controls. We also tested whether antioxidants can decrease systemic ROS in flies. As shown in Figure 4, flies overexpressing Yki generated higher amounts of ROS as compared to the control flies. Yki-overexpressing flies presented an increased rate as well as the total amount of ROS (Figure 4B). Feeding flies with GSH reduced the ROS generation, which was comparable to control flies (Figure 4). We further looked at the tissue level and dissected Drosophila larval hearts from the same genotypes fed with antioxidants (Supplemental Figure S8). In the agreement in Figure 4, where systemic ROS was reduced after feeding flies with antioxidants, we observed reduced ROS staining by DHE in CoQ-fed GMR-Yki flies compared to GMR-Yki on regular media (control).

In order to corroborate our ROS data from flies, we also tested ROS production in mitochondria isolated from mice. We measured ROS generation from cardiac tissues from control and tumor-bearing mice. Similar to what we observed in Drosophila, we detected an increase in ROS production in tumor-bearing mice (Supplemental Figure S9). The rate of ROS production was statistically significant in tumor-bearing mice (Supplemental Figure S9A,B) while total ROS showed an increasing trend but was not statistically significant. These results indicate a ROS-based cardiac dysfunction in the hearts of tumor-bearing mice.

Overall, our results indicate that tumors can have an indirect effect on cardiac function. One of the pathways through which this can happen is through systemic ROS. We hypothesize that the increase in systemic ROS contributes to increased oxidative stress in cardiac tubes, resulting in cardiac dysfunction. Feeding of selective antioxidants to flies overexpressing oncogenes helps to reduce ROS and improve cardiac function.

## 4. Discussion

Our study demonstrates that tumor-induced cardiac dysfunction is evident in mice and *Drosophila melanogaster* model systems to varying degrees depending on the type and location of tumor development. We have shown the occurrence of this phenotype by using induced tumors in mice and overexpressing oncogenes in Drosophila. It is intriguing that not all pathways we tested in Drosophila showed the same phenotype but varied in degree and kind. While most pathways increased heart rate and decreased cardiac ejection fraction and fractional shortening, some pathways, such as JNK (Hep^Act^), showed an increase in cardiac output, while others showed a decrease (PI3K). This emphasizes the importance of the evaluation and prognosis of cardiac dysfunctions related to cancer in an oncogene-specific manner.

We also showed that a similar dysfunctional phenotype can be observed in mice with 67NR breast cancer cell line-induced tumors (that are known to harbor several mutations such- ER-alpha positive, p53 null, etc. [33]), where we observed reduced EF and FS in tumor-bearing mice. Our results are in coherence with previously published work on other cancer models [13]. The development of heart dysfunction in C26 adenocarcinoma and B16F12 melanoma mouse models has been well characterized [12,13,34]. Recent clinical studies on cancer patient populations have also reported cardiac defects in treatment-naïve patients. Kazemi-Bajestani et al. observed that in a cachexia clinical trial of 70 patients with non-small cell carcinoma, 7 displayed reduced LVEF, and 10 had diastolic dysfunction with preserved LVEF [8]. The same group conducted another study where it was observed that cancer patients had significantly reduced left ventricular mass [35]. These studies show that prior to the onset of cachexia and any sort of cancer treatment, tumors can have their own effect on cardiac tissue unrelated to the therapeutic agents or systemic muscle loss. This systemic effect caused by cancer cells could be an important consideration prior to treating cancer patients in order to avoid unfavorable cardiac outcomes post-treatment. Our tumor-bearing mice had comparable body masses to their control counterparts, which indicates that they were not undergoing cachexia at the time of their cardiac function measurements. Additionally, the mice model we have used is not a severe cachexia model. This reinforces the central point of this study that tumors initiate cardiac dysfunction and also indicates that this can even occur prior to the onset of cachexia. It is to be noted that previous studies have implicated oxidative stress in cachectic heart muscles in mouse but our study shows that cardiac phenotypes precede cachexia [13].

ROS has been implicated in causing cardiac dysfunction in our current work using the *D. melanogaster* model. An increase in ROS levels observed in hearts with dysfunction was evident in cachectic hearts, strongly indicating a role in the development of cardiac dysfunction. Gomes et al. illustrated that increasing oxidative stress in a culture of myotubes increased the major components of the protein degradation pathway [36]. It was also demonstrated that oxidative damage in cardiomyocytes is associated with cancer-induced cardiac atrophy [13]. It was revealed that Xanthine oxidase expression was increased while SOD activity was reduced in the heart, implying that this could be majorly contributing to increased ROS in cardiac atrophy [13]. Increased ROS levels have been previously found to trigger the ubiquitin-proteasome system and increase muscle atrophy via the induction of E3 ligases atrogin-1 and MuRF-1 [37,38].

ROS production and oxidative stress are known to contribute to several cardiac disorders such as hypertrophy, heart failure, ischemia-reperfusion injury and cardiomyopathies [39]. All our experiments attempting to measure ROS indicate a trend in increased oxidative stress in a systemic manner in the presence of a tumor. This shows that ROS is at least one of the major candidates from the tumors to the heart that is causing cardiac dysfunction. We observed this increased oxidative stress not only in the Drosophila model system but also in mice. Although there was no increase in total ROS production in tumor-bearing mice, we saw a significant increase in the rate of ROS production. However, there is a trend of increasing total ROS as well in tumor-bearing mice indicating that ROS does increase in cardiac tissue due to a distant tumor. It is intriguing that supplementation of anti-oxidants by food can bring about an effect on cardiac function, restoring it back to wild-type levels. We have tried several anti-oxidants and some of them, such as GSH, CoQ, and vitamin E, have better results than others (vitamin C) again indicating the importance of choosing the right anti-oxidant for therapy. It would be interesting to conduct these studies in mice and higher animals in future studies. Furthermore, our experiments have only dealt with the Yorkie pathway in *Drosophila* for the scope of this paper, but it would be intriguing for future studies to focus on other pathways and examine if the same antioxidants could be effective in flies and other model systems.

## 5. Conclusions

In summary, this is the first study to dwell on the direct effect of cancers on cardiac dysfunction in a genetic model. Our studies for the first time indicate the importance of treatment for possible heart problems that can occur with certain cancers, so that they do not become the burdening cause of severe cardiac dysfunction-related complications, post-cancer therapy. We also showed that deregulation of different oncogenic pathways causes specific systemic effects on the heart and they need to be understood at the molecular level, in order to design specific and effective treatment. Our observation that cardiac dysfunction precedes cachexia suggests that there is an early effect of tumors on cardiac tissues which triggers cardiac dysfunction. At least in case of the Yki pathway that we have studied in detail here, ROS is the mediator of such effects. Further studies are to be carried out in future in higher model organisms in a pathway specific manner to understand how specific oncogenes affect cardiac function.

## Figures and Tables

**Table 1:** Quantification of cardiac function in groups of flies with systemic overexpression of various oncogenes.

	Ejection Fraction	Fractional Shortening	Stroke Volume	Heart Rate	Cardiac Output
Yki	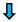	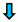	NC	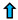	NC
RasV12	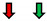	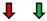	NC	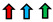	NC
Pi3K	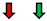	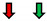	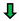	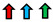	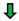
Hep Act	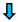	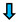	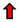	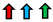	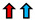

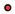
 Dpp Gal4; 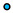 GMR Gal4; 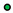 Eyless Gal4; NC: No Change.

## Data Availability

All the data is available within the article.

## Supplementary Materials

The following are available online at https://www.mdpi.com/article/10.3390/antiox10081299/s1, Supplementary figures, Figure S1: Cardiac function of third instar larvae overexpressing PI3K using dpp, GMR, and ey Gal4 drivers. Figure S2: Cardiac function of third instar larvae overexpressing RasV12 using dpp, GMR, and ey Gal4 drivers. Figure S3: Cardiac function of larvae overexpressing HepAct using dpp, GMR, and ey Gal4 drivers. Figure S4: Thickness of pericardin fibers around the cardiac tube in larvae overexpressing oncogenes. Figure S5: Heart function of 7 day old flies overexpressing Yki using GMR-Gal4 driver, with CoQ10 supplementation from day 1–7. Figure S6: Cardiac function of 7-day-old flies overexpressing Yki using GMR-Gal4 driver, with Vitamin E supplementation from day 1–7. Figure S7: Cardiac function of 7-day-old flies overexpressing Yki using GMR-Gal4 driver, with Vitamin C supplementation from day 1–7. Figure S8: Antioxidants reduce ROS in the cardiac tube. Cardiac tubes were dissected from flies fed with regular media or CoQ10.

## References

[1] Sung H., Ferlay J., Siegel R.L., Laversanne M., Soerjomataram I., Jemal A., Bray F. (2021). Global Cancer Statistics 2020: GLOBOCAN Estimates of Incidence and Mortality Worldwide for 36 Cancers in 185 Countries. CA Cancer J. Clin..

[2] Murphy S.L., Xu J., Kochanek K.D., Arias E. (2018). Mortality in the United States, 2017.

[3] von Haehling S., Anker S.D. (2014). Prevalence, incidence and clinical impact of cachexia: Facts and numbers-update 2014. J. Cachexia Sarcopenia Muscle.

[4] von Haehling S., Anker S.D. (2010). Cachexia as a major underestimated and unmet medical need: Facts and numbers. J. Cachexia Sarcopenia Muscle.

[5] Perez I.E., Taveras Alam S., Hernandez G.A., Sancassani R. (2019). Cancer Therapy-Related Cardiac Dysfunction: An Overview for the Clinician. Clin. Med. Insights Cardiol..

[6] Hamo C.E., Bloom M.W. (2019). Chronic Treatment with Multi-Kinase Inhibitors Causes Differential Toxicities on Skeletal and Cardiac Muscles. Cancers.

[7] Hamo C.E., Bloom M.W. (2015). Getting to the Heart of the Matter: An Overview of Cardiac Toxicity Related to Cancer Therapy. Clin. Med. Insights Cardiol..

[8] Kazemi-Bajestani S.M.R., Becher H., Butts C., Basappa N.S., Smylie M., Joy A.A., Sangha R., Gallivan A., Chu Q., Baracos V.E. (2019). Undiagnosed cardiac deficits in non-small cell carcinoma patients in the candidate population for anti-cachexia clinical trials. Support. Care Cancer.

[9] Zheng Y., Chen H., Li X., Sun Y. (2016). Pay attention to cardiac remodeling in cancer cachexia. Support. Care Cancer.

[10] Kazemi-Bajestani S.M., Becher H., Fassbender K., Chu Q., Baracos V.E. (2014). Concurrent evolution of cancer cachexia and heart failure: Bilateral effects exist. J. Cachexia Sarcopenia Muscle.

[11] Barkhudaryan A., Scherbakov N., Springer J., Doehner W. (2017). Cardiac muscle wasting in individuals with cancer cachexia. ESC Heart Fail..

[12] Devine R.D., Bicer S., Reiser P.J., Velten M., Wold L.E. (2015). Metalloproteinase expression is altered in cardiac and skeletal muscle in cancer cachexia. Am. J. Physiol. Heart Circ. Physiol..

[13] Hinch E.C., Sullivan-Gunn M.J., Vaughan V.C., McGlynn M.A., Lewandowski P.A. (2013). Disruption of pro-oxidant and antioxidant systems with elevated expression of the ubiquitin proteosome system in the cachectic heart muscle of nude mice. J. Cachexia Sarcopenia Muscle.

[14] Pietzsch S., Ricke-Hoch M., Stapel B., Hilfiker-Kleiner D. (2019). Modulation of cardiac AKT and STAT3 signalling in preclinical cancer models and their impact on the heart. Biochim. Biophys. Acta Mol. Cell Res..

[15] Caygill E.E., Brand A.H. (2016). The GAL4 System: A Versatile System for the Manipulation and Analysis of Gene Expression. Methods Mol. Biol..

[16] Pyter L.M., Suarez-Kelly L.P., Carson W.E., Kaur J., Bellisario J., Bever S.R. (2017). Novel rodent model of breast cancer survival with persistent anxiety-like behavior and inflammation. Behav. Brain Res..

[17] Patel N.H., Johannesen J., Shah K., Goswami S.K., Patel N.J., Ponnalagu D., Kohut A.R., Singh H. (2018). Inhibition of BKCa negatively alters cardiovascular function. Physiol. Rep..

[18] Chaudhury A., Wanek A., Ponnalagu D., Singh H., Kohut A. (2021). Use of Speckle Tracking Echocardiography to Detect Induced Regional Strain Changes in the Murine Myocardium by Acoustic Radiation Force. J. Cardiovasc. Imaging.

[19] Kohut A., Patel N., Singh H. (2016). Comprehensive echocardiography assessment of the right ventricle in murine models. J. Cardiovasc. Ultrasound.

[20] Lam A., Karekar P., Shah K., Hariharan G., Fleyshman M., Kaur H., Singh H., Gururaja Rao S. (2018). Drosophila Voltage-Gated Calcium Channel alpha1-Subunits Regulate Cardiac Function in the Aging Heart. Sci. Rep..

[21] Goswami S.K., Ponnalagu D., Hussain A.T., Shah K., Karekar P., Gururaja Rao S., Meredith A.L., Khan M., Singh H. (2018). Expression and Activation of BKCa Channels in Mice Protects against Ischemia-Reperfusion Injury of Isolated Hearts by Modulating Mitochondrial Function. Front. Cardiovasc. Med..

[22] Gardiner B., Dougherty J.A., Ponnalagu D., Singh H., Angelos M., Chen C.A., Khan M., Berliner L.J., Parinandi N.L. (2020). Measurement of Oxidative Stress Markers In Vitro Using Commercially Available Kits. Measuring Oxidants and Oxidative Stress in Biological Systems.

[23] Singh H., Lu R., Rodriguez P.F., Wu Y., Bopassa J.C., Stefani E., Toro L. (2012). Visualization and quantification of cardiac mitochondrial protein clusters with STED microscopy. Mitochondrion.

[24] Ponnalagu D., Gururaja Rao S., Farber J., Xin W., Hussain A.T., Shah K., Tanda S., Berryman M., Edwards J.C., Singh H. (2016). Molecular identity of cardiac mitochondrial chloride intracellular channel proteins. Mitochondrion.

[25] Lee A., Lin A., Shah K., Singh H., Miller V., Gururaja Rao S. (2016). Optimization of Non-Thermal Plasma Treatment in an In Vivo Model Organism. PLoS ONE.

[26] Tian M., Asp M.L., Nishijima Y., Belury M.A. (2011). Evidence for cardiac atrophic remodeling in cancer-induced cachexia in mice. Int. J. Oncol..

[27] Saavedra P., Perrimon N. (2019). Drosophila as a Model for Tumor-Induced Organ Wasting. Adv. Exp. Med. Biol..

[28] Oh H., Irvine K.D. (2010). Yorkie: The final destination of Hippo signaling. Trends Cell Biol..

[29] Huang J., Wu S., Barrera J., Matthews K., Pan D. (2005). The Hippo signaling pathway coordinately regulates cell proliferation and apoptosis by inactivating Yorkie, the Drosophila Homolog of YApp. Cell.

[30] Dong J., Feldmann G., Huang J., Wu S., Zhang N., Comerford S.A., Gayyed M.F., Anders R.A., Maitra A., Pan D. (2007). Elucidation of a universal size-control mechanism in Drosophila and mammals. Cell.

[31] Nagaraj R., Gururaja-Rao S., Jones K.T., Slattery M., Negre N., Braas D., Christofk H., White K.P., Mann R., Banerjee U. (2012). Control of mitochondrial structure and function by the Yorkie/YAP oncogenic pathway. Genes Dev..

[32] Tsutsui H., Kinugawa S., Matsushima S. (2011). Oxidative stress and heart failure. Am. J. Physiol. Heart Circ. Physiol..

[33] Johnstone C.N., Smith Y.E., Cao Y., Burrows A.D., Cross R.S., Ling X., Redvers R.P., Doherty J.P., Eckhardt B.L., Natoli A.L. (2015). Functional and molecular characterisation of EO771.LMB tumours, a new C57BL/6-mouse-derived model of spontaneously metastatic mammary cancer. Dis. Models Mech..

[34] Thackeray J.T., Pietzsch S., Stapel B., Ricke-Hoch M., Lee C.W., Bankstahl J.P., Scherr M., Heineke J., Scharf G., Haghikia A. (2017). Insulin supplementation attenuates cancer-induced cardiomyopathy and slows tumor disease progression. JCI Insight.

[35] Kazemi-Bajestani S.M.R., Becher H., Butts C., Basappa N.S., Smylie M., Joy A.A., Sangha R., Gallivan A., Kavsak P., Chu Q. (2019). Rapid atrophy of cardiac left ventricular mass in patients with non-small cell carcinoma of the lung. J. Cachexia Sarcopenia Muscle.

[36] Gomes-Marcondes M.C., Tisdale M.J. (2002). Induction of protein catabolism and the ubiquitin-proteasome pathway by mild oxidative stress. Cancer Lett..

[37] Razeghi P., Baskin K.K., Sharma S., Young M.E., Stepkowski S., Essop M.F., Taegtmeyer H. (2006). Atrophy, hypertrophy, and hypoxemia induce transcriptional regulators of the ubiquitin proteasome system in the rat heart. Biochem. Biophys. Res. Commun..

[38] Bjorklund G., Dadar M., Aaseth J., Chirumbolo S., Pen J.J. (2019). Cancer-associated cachexia, reactive oxygen species, and nutrition therapy. Curr. Med. Chem..

[39] Peoples J.N., Saraf A., Ghazal N., Pham T.T., Kwong J.Q. (2019). Mitochondrial dysfunction and oxidative stress in heart disease. Exp. Mol. Med..

