# Supplementary material for: Tumor-Induced Cardiac Dysfunction: A Potential Role of ROS
Source: Antioxidants (Basel). 2021 Aug 18;10(8):1299. doi: 10.3390/antiox10081299 (PMC8389295; doi:10.3390/antiox10081299)
Supplement: Supplementary file 1 [file antioxidants-10-01299-s001.zip › antioxidants-1312526-supplementary.pdf]

Supplemental files

Tumor-induced cardiac dysfunction: a potential role of ROS

Priyanka Karekar[1], Haley N. Jansen[1], Kathryn L.G. Russart[2], Devasena Ponnalagu[1], Sarah Seeley[3], Shridhar Sanghvi[1], Sakima A. Smith[4], Leah M. Pyter[2], Harpreet Singh[1*], Shubha Gururaja Rao[1, 3*]

[1]Department of Physiology and Cell Biology, The Ohio State University, Columbus OH 43210

[2]Institute for Behavioral Medicine Research, Departments of Psychiatry and Behavioral Health & Neuroscience, The Ohio State University, Columbus OH 43210

[3]Department of Pharmaceutical and Biomedical Sciences, Raabe College of Pharmacy, Ohio Northern University, Ada, OH 45810

[4]Division of Cardiovascular Medicine, Ohio State University Wexner Medical Center, Columbus, OH, USA

* Co-corresponding authors

Harpreet.singh@osumc.edu

s-gururajarao@onu.edu

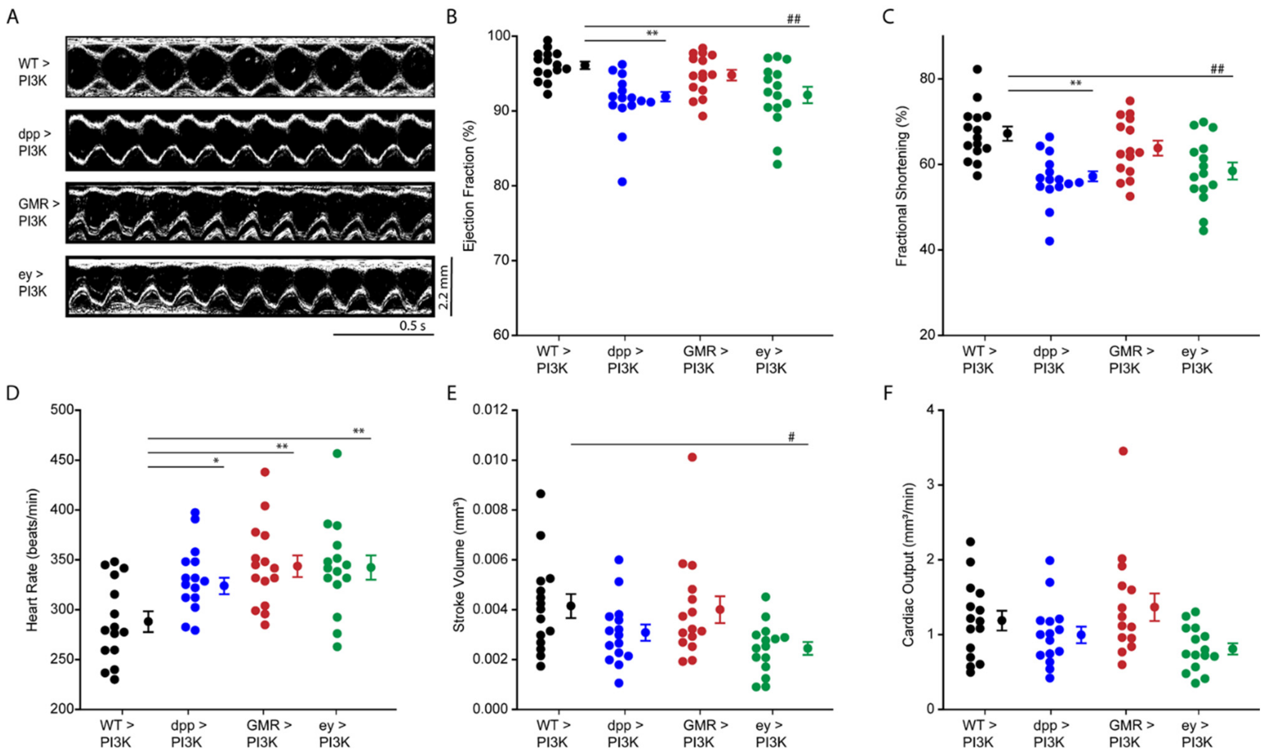

Supplemental Figure S1: Cardiac function of third instar larvae overexpressing PI3K using dpp, GMR, and ey Gal4 drivers. PI3K overexpressed in dpp, GMR, and eyeless Gal4 drivers show varying degrees of cardiac dysfunction in *D. melanogaster* larvae. **A**. OCT images of flies. dpp>PI3K resulted in reduced EF and FS (**B, C**). ey>PI3K showed the most cardiac dysfunction, with reduced EF, FS, and SV (**B, C, E**). Heart rate was found to be significantly higher in overexpression using all three drivers (**E**). However, PI3K overexpression did not cause a significant reduction in cardiac output in any groups (**F**). #p < 0.05; *p < 0.01; ##p < 0.005; **p < 0.001, n≥10. Statistical significance was calculated by the Holm-Sidak test (multiple comparisons to control), and the Student-Newman-Keuls test (multiple pairwise comparisons).

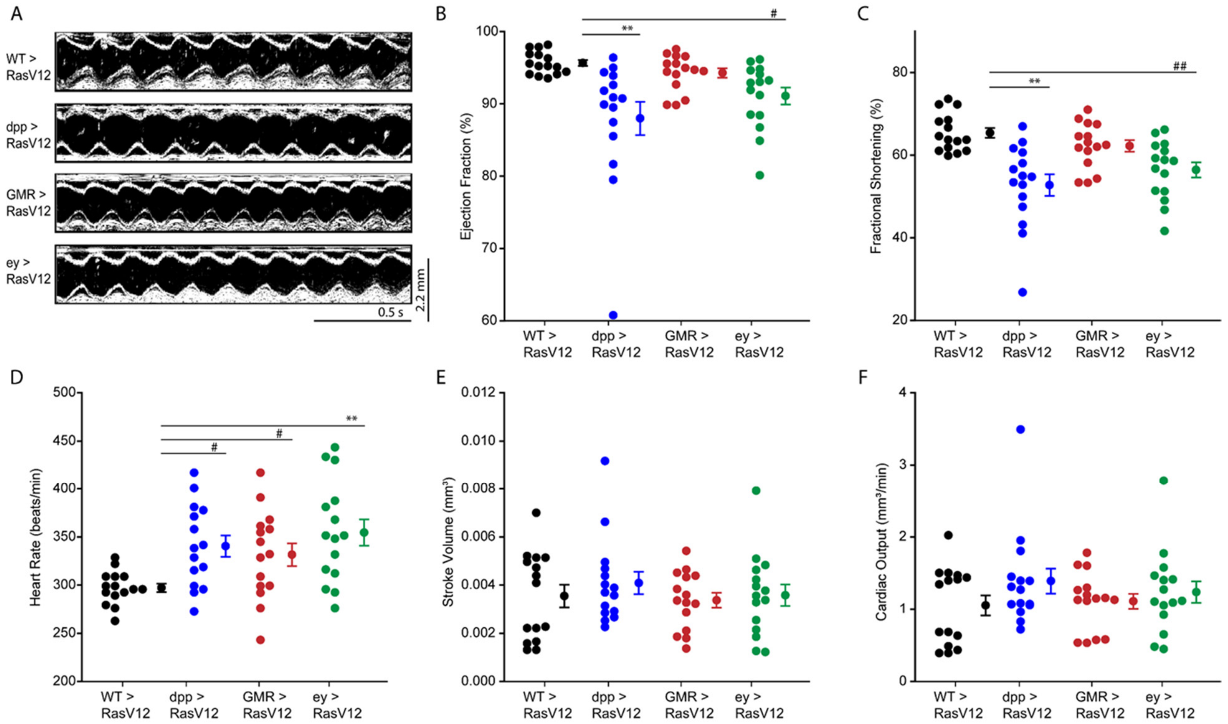

Supplemental Figure S2: Cardiac function of third instar larvae overexpressing RasV12 using dpp, GMR, and ey Gal4 drivers. RasV12 overexpression caused cardiac dysfunction in 3rd instar larvae. **A**. OCT images of flies. dpp>RasV12 and ey>RasV12 caused a reduction in EF and FS **(B, C).** Heart rate was increased significantly with all three drivers compared to WT control **(D),** but there were no changes in SV and CO **(E, F).** #p < 0.05; ##p < 0.005; **p < 0.001, n≥10. Statistical significance was calculated by the Holm-Sidak test (multiple comparisons to control), and the Student-Newman-Keuls test (multiple pairwise comparisons).

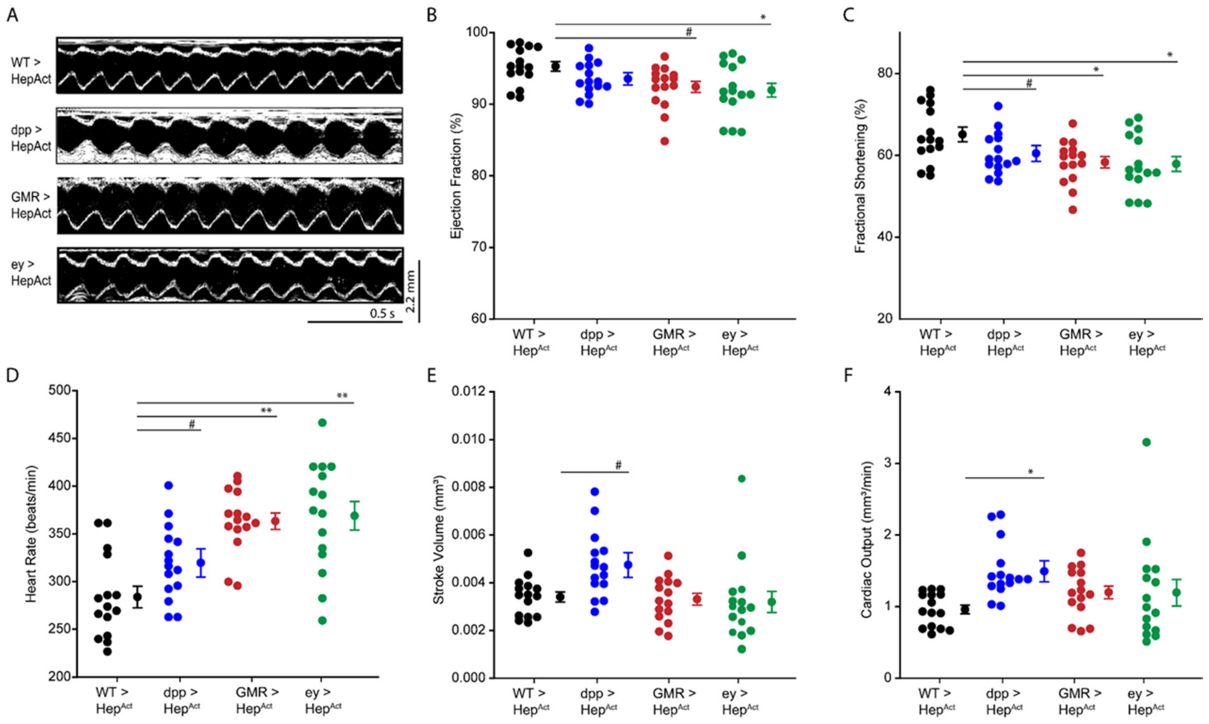

Supplemental Figure S3: Cardiac function of larvae overexpressing Hep^{Act} using dpp, GMR, and ey Gal4 drivers. A. OCT images of flies. **B.** EF obtained from OCT traces. HepAct overexpression results in significant cardiac dysfunction. dpp>Hep^{Act} shows a significant reduction in FS and increases in HR (**C, D**). Interestingly, we observe an increase in stroke volume and cardiac output in this group, as compared to the control. GMR>HepAct larvae exhibit lowered EF and FS, and increased HR (**B, C, D**). ey>HepAct shows the most dysfunction, with increased EF and FS, and increased HR. #p < 0.05; *p< 0.01; ##p < 0.005; **p< 0.001, n≥10. Statistical significance was calculated by the Holm-Sidak test (multiple comparisons to control), and the Student-Newman-Keuls test (multiple pairwise comparisons).

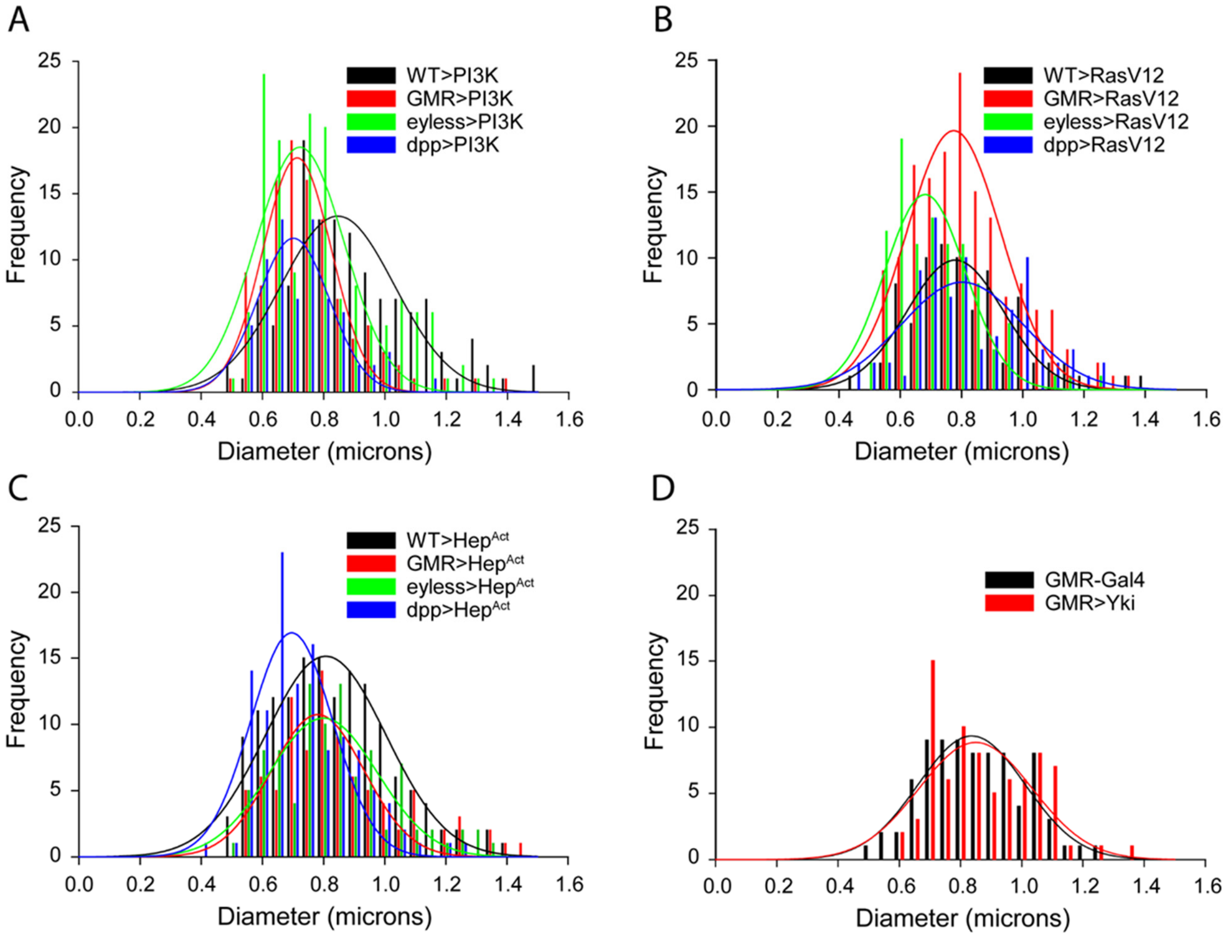

Supplemental Figure S4: Thickness of pericardin fibers around the cardiac tube in larvae overexpressing oncogenes. Frequency histograms of pericardin fiber thickness around the cardiac tube in *D. melanogaster* hearts. No significant differences were observed in pericardin fiber thickness of larvae overexpressing oncogenes PI3K (**A**), RasV12 (**B**), Hep^{Act} (**C**), and Yki (**D**) using drivers dpp, GMR, and eyeless Gal4, indicating that overexpressing these oncogenes did not cause cardiac fibrosis, $n \geq 10$. Statistical significance was calculated by the Holm-Sidak test (multiple comparisons to control), and the Student-Newman-Keuls test (multiple pairwise comparisons).

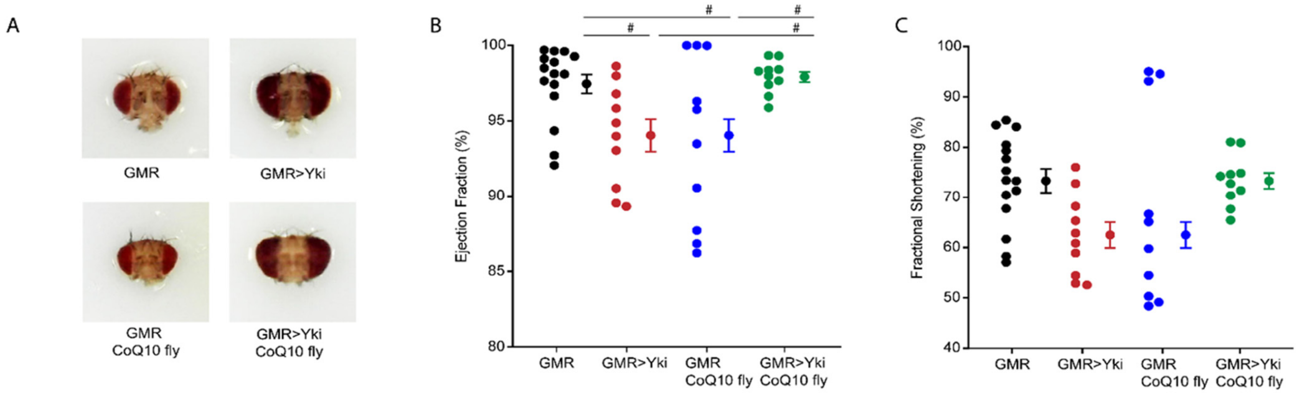

Supplemental Figure S5: Heart function of 7 day old flies overexpressing Yki using GMR-Gal4 driver, with CoQ10 supplementation from day 1-7. (A) Images of 7 day old D. melanogaster eyes with and without tumor and/or CoQ10 supplementation. **(B),** and **(C),** show EF, and FS, respectively **(**n=10 each**)**. #p < 0.05**.** Statistical significance was calculated by the Holm-Sidak test (multiple comparisons to control), and the Student-Newman-Keuls test (multiple pairwise comparisons).

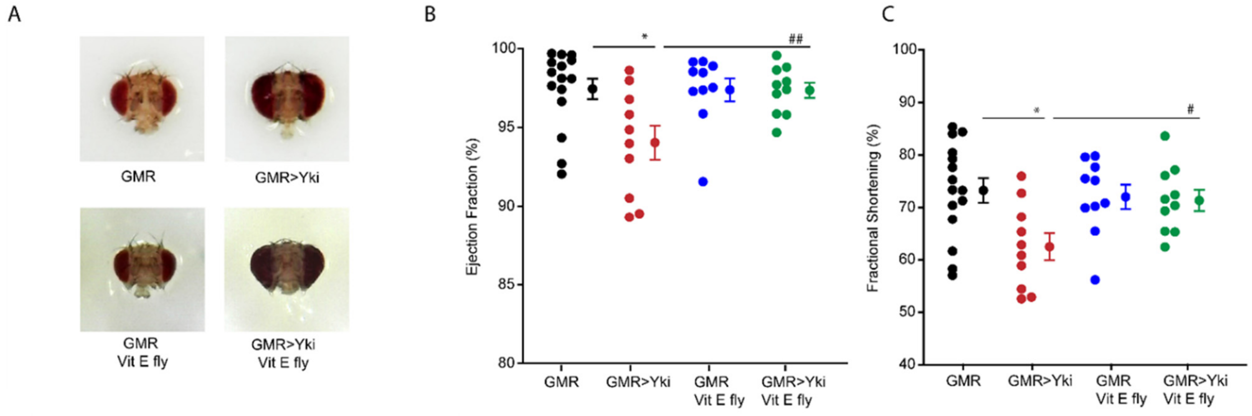

Supplemental Figure S6: Cardiac function of 7-day-old flies overexpressing Yki using GMR-Gal4 driver, with Vitamin E supplementation from day 1-7. Vitamin E supplementation did not cause a reduction in tumor **(A).** Ejection fraction **(B)** and fractional shortening **(C)** were improved in GMR>Yki flies supplemented with Vit E**.** #p < 0.05; *p < 0.01; ##p < 0.005, n≥10. Statistical significance was calculated by the Holm-Sidak test (multiple comparisons to control), and the Student-Newman-Keuls test (multiple pairwise comparisons).

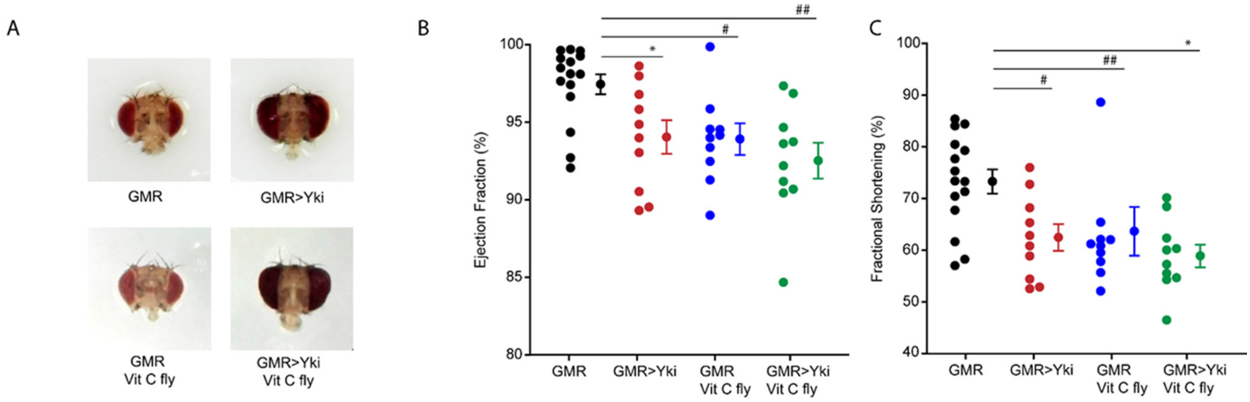

Supplementary Figure S7: Cardiac function of 7-day-old flies overexpressing Yki using GMR-Gal4 driver, with Vitamin C supplementation from day 1-7. Vitamin C supplementation did not cause a reduction in tumor **(A).** Please note that the tumor in GMR>Yki is not changing with Vit C **(A).** There is no significant rescue in cardiac parameters - ejection fraction **(B),** fractional shortening **(C),** heart rate **(D),** stroke volume **(E),** and cardiac output **(F)**. #$p < 0.05$; *$p < 0.01$; ##$P < 0.005$; **$p < 0.001$, $n \geq 10$. Statistical significance was calculated by the Holm-Sidak test (multiple comparisons to control), and the Student-Newman-Keuls test (multiple pairwise comparisons).

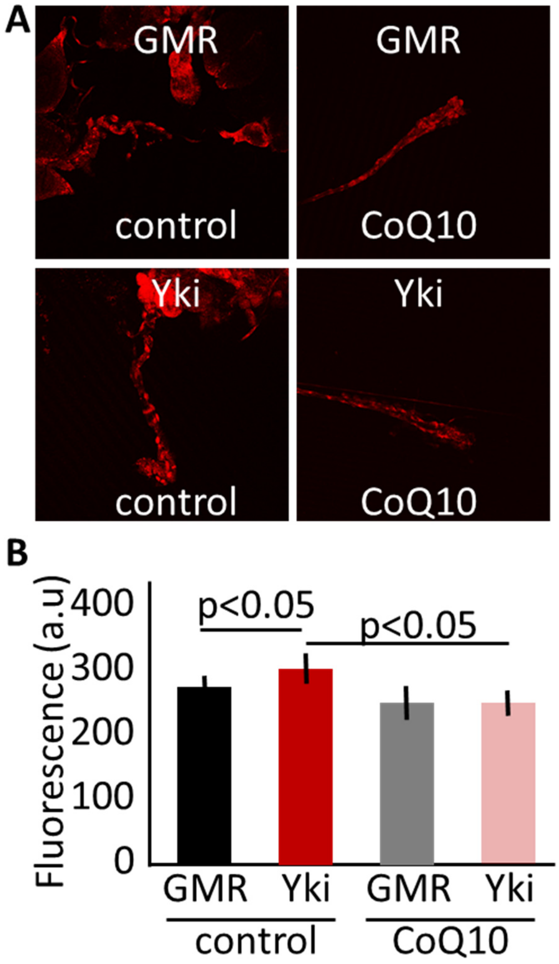

Supplemental Figure S8. Antioxidants reduce ROS in the cardiac tube. Cardiac tubes were dissected from flies fed with regular media or CoQ10. Cardiac tubes were stained with DHE and imaged with a confocal microscope. **A.** Confocal images of cardiac tubes of flies fed with regular food or food supplemented with antioxidants (CoQ10). **B.** Quantification of DHE stained ROS in cardiac tubes**.** n=5-8 flies. Statistical significance was calculated by Student’s t-test.

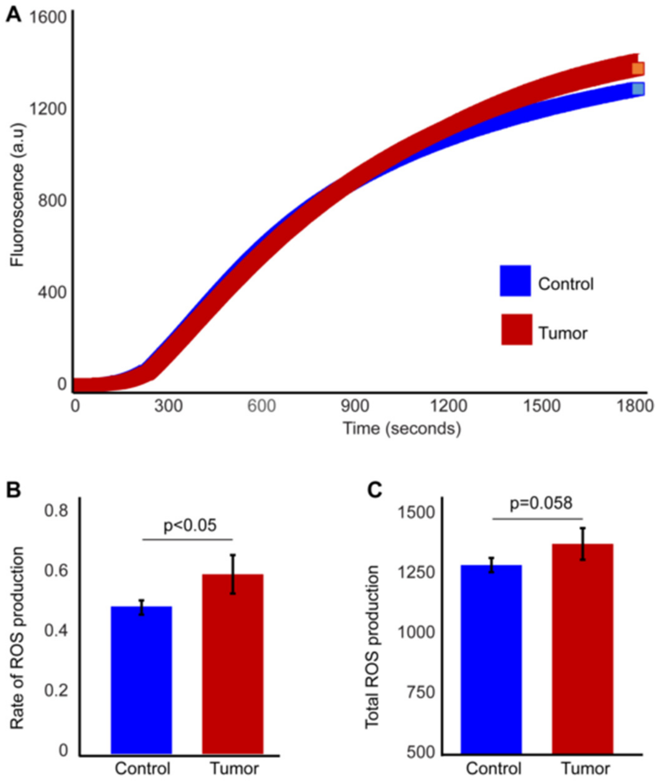

Supplemental Figure S9. Tumor-bearing mice hearts show an increased rate of mitochondrial ROS generation. ROS was measured in hearts of isolated mitochondria from control and tumor-bearing mice. **A.** Graph representing ROS production for 30 mins after addition of isolated mitochondria from control (blue), tumor (red). **B.** Bar graph representing a rate of ROS production, tumor-bearing mice have a significantly high rate of ROS production #p < 0.05; n≥4. **C.** Total ROS generation calculated from **A,** there is a trend for increased total ROS in tumor mice but it is not statistically significant n≥4**.** (statistical significance was calculated by Student's t-test).
